# A Decolonizing Approach in Population Health Research: Examining the Association between the federal maternal evacuation policy on Maternal and Child outcomes in First Nation (FN) Communities in Manitoba

**DOI:** 10.23889/ijpds.v7i3.1825

**Published:** 2022-08-25

**Authors:** Wanda Phillips-Beck, Nathan Nickel, Marni Brownell, Josée Lavoie, Shultz Annette, Jaime Cidro

**Affiliations:** 1 First Nation Health and Social Secretariat of Manitoba and University of Manitoba; 2 Manitoba Centre for Health Policy, University of Manitoba; 3 Community Health Sciences, University of Manitoba; 4 College of Nursing, University of Manitoba; 5 University of Winnipeg

## Objectives

Responding to the Truth and Reconciliation Commission of Canada’s (TRC) Call to Action #19 to close the gap in maternal/child outcomes, the goal of this study was to provide a baseline for select outcomes and demonstrate how an Indigenous/decolonizing framework can be applied to population health research involving Indigenous people.

## Approach

This retrospective cohort study was embedded within a decolonizing and Indigenous framework. Data extracted from administrative data housed at the Manitoba Centre for Health Policy was utilized to create a cohort of low-risk women residing in FN communities delivering a baby between 2005-2015. Two groups of mother/child dyads were compared: those evacuated for birth and those who were not required to leave home. Data were analyzed to assess the association between the evacuation policy on health outcomes.

## Results

Decolonizing and Indigenous frameworks are feasible, essential, and necessary in population health research involving Indigenous people. This methodology does not detract from scientific rigor. In keeping with Indigenous methodology, Knowledge Keepers and a Grandmother Advisor informed the research from the onset, including insightful dialogue about the study findings. Using such an approach, this study generated evidence that the present-day OFC policy continues to harm Indigenous women, families, and communities. The OFC policy is associated with increased odds of inadequate PNC (OR 1.64 1.51, 1.79 CI) and small for gestational age births (OR 1.25 1.02, 1.50 CI) and decreased breastfeeding initiation (OR 0.55 0.50, 0.61 CI) and maternal psychological distress diagnoses (OR .43 0.36, 0.51), after adjusting for various confounders.

## Conclusion

This study documented a journey of an Anishinaabekwe in the space where western and Indigenous methodologies met. In answering the TRC call to improve maternal and infant outcomes, epidemiological and population health research requires epistemological frameworks that adequately incorporate the voices and realities of Indigenous people's lives while remaining scientifically rigorous.

